# The Antifungal Activity of Functionalized Chitin Nanocrystals in Poly (Lactid Acid) Films

**DOI:** 10.3390/ma10050546

**Published:** 2017-05-18

**Authors:** Asier M. Salaberria, Rene H. Diaz, María A. Andrés, Susana C.M. Fernandes, Jalel Labidi

**Affiliations:** 1 Biorefinery Processes Research Group, Department of Chemical and Environmental Engineering, Faculty of Engineering, University of the Basque Country (UPV/EHU), Pza. Europa 1, 20018 Donostia-San Sebastian, Spain; asier.martinez@ehu.eus (A.M.S.); renealexander.herrera@ehu.eus (R.H.D.), marian.andres@ehu.eus (M.A.A.); 2 CNRS/Université de Pau et des Pays de l’Adour, Institut des Sciences Analytiques et de Physico-Chimie pour l’Environnement et les Materiaux, UMR 5254, 2 Av. Pdt Angot, 64053 Pau, France

**Keywords:** poly(lactic acid), chitin nanocrystals, nanocomposites, barrier properties, fungal inhibition

## Abstract

As, in the market, poly (lactic acid) (PLA) is the most used polymer as an alternative to conventional plastics, and as functionalized chitin nanocrystals (CHNC) can provide structural and bioactive properties, their combination sounds promising in the preparation of functional nanocomposite films for sustainable packaging. Chitin nanocrystals were successfully modified via acylation using anhydride acetic and dodecanoyl chloride acid to improve their compatibility with the matrix, PLA. The nanocomposite films were prepared by extrusion/compression approach using different concentrations of both sets of functionalized CHNC. This investigation brings forward that both sets of modified CHNC act as functional agents, i.e., they slightly improved the hydrophobic character of the PLA nanocomposite films, and, very importantly, they also enhanced their antifungal activity. Nonetheless, the nanocomposite films prepared with the CHNC modified with dodecanoyl chloride acid presented the best properties.

## 1. Introduction

Among the available biodegradable polymers in the market, poly(lactic acid) (PLA) is the most used as a sustainable alternative to conventional plastics [1,2,3,4]. Lactic acid can be produced through microbial fermentation of agricultural by-products, namely corn, sugar beets, wheat and potato starch [1,4,5]; and PLA, which is an aliphatic thermoplastic polyester, can be obtained by polymerization of lactic acid monomers (hydroxyl carboxylic acids). Because of its properties, namely good mechanical strength and stiffness, UV stability and gloss, PLA is often comparable to polystyrene and polypropylene. Thus, increasing attention in the design and development PLA-based materials for food packaging, biomedical devices and automotive industry has been observed [6,7,8].

Similar to petrochemical derivatives, several strategies have been developed to improve the structural and/or functional properties of the polymer-based materials from renewable resources, including the incorporation of fillers. Natural fillers, such as nanofibers and nanocrystals from cellulose and chitin, and nanocrystals from starch are gaining considerable interest because of their abundance, chemical structure, low toxicity and biodegradability [9,10,11,12,13,14,15,16]. Moreover, chitin nanofillers provide antifungal or antibacterial properties [17,18,19].

Chitin is a crystalline high molecular weight linear polysaccharide consisting of β-(1 → 4)-2-acetamido-2-deoxy-d-glucopyranose repeating units, which is found in the crustacean shells, insect cuticles and in the cell walls of fungi, yeast and green algae. It occurs as a highly-organized micro- and nano-fibril structure increasing from the nanometer to the millimeter scale [20]. These fibrils form highly crystalline regions and disordered (amorphous) regions that can be turned in nanocrystals via top-down methods such as acid hydrolyses by dissolving the amorphous regions [18,21,22,23,24].

Nonetheless, the use of natural nanofillers, including nanochitin, on composite materials presents some drawbacks, in particular, their high polar character is responsible for a very low interfacial compatibility with common thermoplastic matrices, including PLA. Consequently, different approaches have been employed to overcome this issue [25,26]. Among them, the surface acylation of the natural nanofillers by the introduction of hydrophobic functional groups under heterogeneous conditions seems to be an efficient way to solve the compatibility issue [9,27,28,29,30,31,32].

Thus far, few studies regarding the use of chitin nanofillers as reinforcing agents in PLA matrices have been described in literature [25,26,33,34,35,36]. Following our interest in the preparation of chitin nanofillers-based materials [18,20,21,22,23,37,38], the present study goes a step further than the use chitin nanofillers as reinforcing agents. The aim of this work was to improve the compatibility of chitin nanocrystals with PLA matrix and evaluate their behavior as functional agents for potential use as active food packaging.

## 2. Materials and Methods

### 2.1. Materials

*Cervimunida johni* lobster (known as yellow lobster) wastes in the form of powder were kindly supplied by Antartic Seafood S.A. (Coquimbo, Chile). Poly(lactic acid) (PLA, l-poly-lactide content is ≥99% *w*/*w*) with Mw of 180,000 g mol^−1^ in the form of pellets was kindly provided by Futerro S.A. (Celles, Belgium). Dodecanoyl chloride acid (98.0%) was purchased from Aldrich. Hydrochloric acid (HCl, ACS reagent, 37.0%), sulfuric acid (H_2_SO_4_, ACS reagent, ≥95%), acetic anhydride (Ac_2_O, ACS reagent, ≥98.0%), dimethylformamide (DMF, anhydrous, 99.8%), acetone (analytical standard, ≥99%), ethanol (analytical standard, 70%) and pyridine (ACS reagent, 99%) were purchased by Panreac (Barcelona, Spain). All reagents were used as received with exception of pyridine that was purified by distillation over sodium hydroxide.

### 2.2. Chitin Extraction

α-Chitin (CH) was extracted from yellow lobster wastes according our previous works [18,21,22,23]. Three main steps were applied in the following sequence: deproteinization—removal of proteins in a solution of 2 M NaOH at room temperature for 24 h; demineralization—removal of minerals (CaCO_3_) in a solution of 2 M HCl at room temperature for 3 h; and decolorization—extraction of pigments and lipids using acetone followed by ethanol under reflux for 6 h. Afterwards, α-chitin was filtered and washed with deionized water twice. The resulting α-chitin was dried at 60.0 ± 0.5 °C in an oven overnight. The degree of N-acetylation was found to be 96% by ^13^C-NMR [39].

### 2.3. α-Chitin Nanocrystals Extraction

Chitin nanocrystals (CHNC) were isolated from the obtained α-chitin based on our previous work [18,21,22,23] adapted from the method developed by Paillet et al. [24]. To hydrolyze the amorphous regions of chitin, α-chitin powder was dispersed in a solution of 3 M HCl at 100 ± 2 °C and left for 90 min under vigorous stirring and refluxing. After acid hydrolysis, the obtained suspension was dispersed in deionized water and washed by centrifugation. This procedure was repeated three times. The suspension was then dialyzed changing deionized water every 12 h until pH 6. Then the suspension was subjected to ultrasonic treatment for 10 min (Vibracell 75043 from Bioblock Scientific) to disintegrate chitin nanocrystal aggregates and obtain a homogeneous suspension. Finally, chitin nanocrystals were filtered to get a final suspension of 4 wt % and stored at 4 °C before used. CHNC presented a rod-like morphology with average wide of 60 nm and length of 300 nm. Their degree of acetylation was estimated to be 92% by ^13^C-NMR spectroscopy [39] and crystallinity index to be 89% using the method of Focher et al. [40].

### 2.4. Surface Functionalization of α-Chitin Nanocrystals

#### 2.4.1. Acylation with Acetic Anhydride

**Figure 1:**
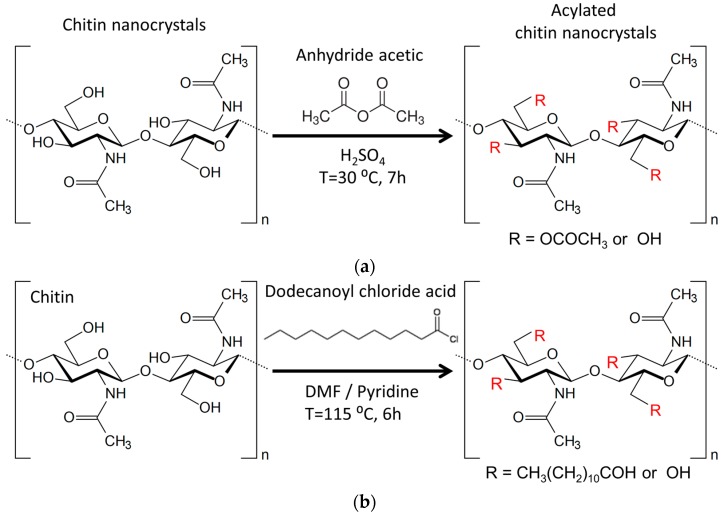
Scheme representing the heterogeneous chemical functionalization of chitin nanocrystals using: anhydride acetic (**a**); and dodecanoyl chloride acid (**b**).

The acylation of CHNC using acetic anhydride and sulfuric acid as catalyst was adapted from the method used by Frisoni et al. [41] (Figure 1a).

Never dried CHNC (6 wt % suspension in water) were submitted to solvent exchange to acetone via ethanol as intermediate solvent, with three dispersion/filtration sequences for each solvent (50 mL), and then in acetic anhydride (50 mL) with two dispersion/filtration sequences for 15 min.

Afterwards, CHNC were dispersed in 15 mL of acetic anhydride containing 3.6 wt % sulfuric acid (0.2 mL) of the dry chitin weight. The reaction mixture was stirred at 30 °C for 7 h. At the end of the reaction, the acylated chitin nanocrystals (C_2_CHNC) were filtered and sequentially washed with acetone and ethanol. To remove residual trace of acetic anhydride and other impurities, the C_2_CHNC were Soxhlet extracted with ethanol overnight and finally dried at 60 °C for 24 h.

#### 2.4.2. Acylation with Dodecanoyl Chloride Acid (Fatty Acid)

The acylation of CHNC using dodecanoyl chloride acid was carried out according the method described by Freire et al. [42] (Figure 1b). Dodecanoyl chloride acid (1 eq. relative to the total OH groups of chitin) was placed in a 250 mL round-bottom flask. DMF (100 mL), pyridine and CHNC (2 g) were then added and maintained under stirring at 115 °C for 6 h. After esterification, the mixture was filtered and washed with DMF, acetone and ethanol. To remove any residual trace of fatty acid, acylated chitin nanocrystals (C_12_CHNC) were Soxhlet extracted with ethanol overnight and finally dried at 60 °C in an oven for 24 h.

### 2.5. Preparation of PLA/C_2_CHNC and PLA/C_12_CHNC Nanocomposite

PLA/C_2_CHNC and PLA/C_12_CHNC nanocomposites were prepared by compression molding approach as following:

(1) PLA pellets were dissolved in chloroform (10 mL g^−1^) into a 250 mL glass beaker.
(2) Different amounts (i.e., 0.5, 1.0 and 2.0 wt %) of each C_2_CHNC and C_12_CHNC sets were added in the different glass beaker.
(3) The mixtures were stirred for 5 min and then left at 30 °C for 1 day to evaporate the solvent.
(4) After solvent evaporation, the PLA/C_2_CHNC and PLA/C_12_CHNC mixtures were extruded using a HAAKE MiniLab micro compounder at 180 ± 2 °C and 120 rpm.
(5) Subsequently, the nanocomposites were molded by compression using a Metrotec press at 190 °C and 50 bar for 1 min, followed by fast cooled.
(6) Finally, the samples were kept at 50% relative humidity (RH) and 25 ± 2 °C for 72 h before being tested.

Samples identification is listed in Table 1.

### 2.6. Physicochemical and Morphological Characterization

Attenuated total reflection Fourier transform infrared spectroscopy (ATR-FTIR) spectra were recorded on a Nicolet Nexus 670 equipped with a KRS-5 crystal of refractive index 2.4 and using an incidence angle of 45°. The spectra were taken in a transmittance mode in the wavenumber range of 750–4000 cm^−1^, with resolution of 4 cm^−1^ and after 128 scan accumulations. The spectra were normalized using the software Omnic.

X-ray diffraction patterns were collected using a Philips X’pert Pro automatic diffractometer using Cu-Kα radiation operating at 40 kV and 40 mA over the angular range of 5 to 40° 2θ (step size = 0.04 and time per step = 353 s) at room temperature. The crystallinity index (C.I.) of chitin nanocrystal samples was calculated as follows (Equation (1)) [40,43]:

(1) $$C.I.\left(\%\right)=[\left(I_{110}-I_{am}/I_{110}\right]\times 100$$

where *I*_110_ is the maximum intensity (arbitrary units) of the 110 crystallographic plane and *I_am_* is the amorphous portion diffraction, which usually is found about 2θ = 12.5–13.5°.

Atomic force microscopy (AFM) images were performed in a Dimension 3100 NanoScope IV (Veeco, Santa Barbara, CA, USA). The images were scanned in tapping mode under ambient conditions using silicon nitride cantilevers having a tip nominal radius of 10 nm at a frequency of 1 Hz. Chitin nanocrystal samples were first well diluted in water and sonicated for 5 min until colloidal suspension (Vibracell 75043 from Bioblock Scientific). Then a drop of the suspension was casted on to a mica piece, which is late subjected to a spin coating equipment (Specialty coating systems INC, Spin Coater model P6700 series) at 2000 rpm for 2 min. The dimensions of nanocrystals were calculated by measuring 100 different nanocrystals.

The light transmittance spectra of the materials were measured using a Shimadzu (Kyoto, Japan) UV-3600 UV-VIS-NIR spectrophotometer. Spectra were recorded at room temperature in steps of 1 nm, in the range 400–700 nm.

Contact angle measurements were performed at room temperature using Dataphysics OCA20 Contact Angle System (Filderstadt, Germany). Three different liquids, with different dispersive and polar surface tensions (water, diiodomethane and ethylene glycol), were used to determine the surface energy of the samples following the Owens and Wendt approach [44]. To perform the measurements, 50 ± 5 mg of each functionalized CHNC were placed into a cylindrical holder and pressed using a hydraulic press to obtain uniform discs (13 mm in diameter and 0.4 mm in thickness). The test was done in triplicate.

### 2.7. Barrier Properties

Water vapor transmission tests of the materials (thickness range from 0.06 to 0.10 ± 0.01 mm) were performed at 30.0 ± 0.2 °C on a gravimetric cell in which a small amount of liquid water (2 mL) was sealed by a membrane. The cell was put on an analytical balance (±10^−5^ g), and the weight loss of the cell, only due to the permeation of the water vapor through the membrane, was followed by a computer connected to the balance. Water activity inside the cell was 1, whereas the downstream average humidity was 33 ± 2% as recorded with a thermohygrometer. Changes in the weight of the cell were recorded as a function of time. The water vapor transmission rate (WVTR) was determined from the following Equation (2):

(2) $$\mathrm{WVTR}\;\left(\frac{g.\mathrm{mm}}{m^{2}.\mathrm{day}}\right)=\frac{m\;.\ell }{A\left(1-a\right)}$$

where WVTR is the water vapor flow passing through the films per unit time; “ℓ” is the thickness of the films; “A” is the area of the films (2.54 cm^2^) exposed to the water vapor flow; and “a” is the relative humidity in the thermostatic system. For each film, WVP measurements were performed at least three times.

### 2.8. Antifungal Activity against Aspergillus Niger

In order to evaluate the anti-fungal activity of the composite films against a common rot of food and indoor environments, the mold fungus *Aspergillus niger* (CBS 554.65) was cultured and incubated aerobically for 72 h at 25 °C in Petri dishes with solid substrate of Malte Extract Agar (MEA, Sharlab). After growing, an aliquot of spores was diluted in Ringer solution (14 mL) and was aseptically inoculated on the MEA surfaces by spray method (with 40 μL of dissolution at 1.21 × 106 Spores mL^−1^).

Finally, 1 cm × 1 cm shaped samples (with an average thickness of 0.08 mm) of each nanocomposite films and PLA films (the latter was used as control) were introduced into the MEA plates to assess the fungal inhibition. Each sample was prepared in triplicate.

After 7 days of incubation at 25 ± 1.5 °C, the films were gently extracted from the agar and washed with Ringer solution (1 mL) to obtain a spore solution. Then samples were vortexed and stained with LPCB taking 20 μL of each sample to count spores concentration with an automated cell counter (Cellometer^®^ Mini Nexcelom Bioscience LLC, Lawrence, MA, USA). The fungal growth inhibition (*FGI*) was calculated as concentration of spores (conidia) per milliliter, according to the Equation (3):

(3) $$FGI\;\left(\%\right)=\frac{C_{g}-T_{g}}{C_{g}}\times 100$$

where *C_g_* is the average concentration in the control samples set (PLA films and fungal dish) and *T_g_* is the average concentration in the treated set (film tested), both expressed as concentration of spores (conidia) per milliliter [18].

### 2.9. Thermal Stability and Mechanical Properties

Thermogravimetric analysis (TGA) assays were carried out in a TGA/SDTA 851 Mettler Toledo instrument. All samples (~8 mg) were heated at a constant rate of 10 °C min^−1^ from 25 to 900 °C under a nitrogen atmosphere of 20 mL min^−1^.

Tensile tests were conducted under ambient conditions using a Material Testing Systems (MTS Insight 10) device using a load cell of 250 N and a deformation rate of 3 mm min^−1^. Ten replicates of each sample (rectangular-shape specimens of 6 × 1 cm^2^ and gauge length of 2 cm) were used to determine the average values of Young’s modulus, tensile strength and elongation at break using MTS TestWorks 4 software (Version 4.09A, MTS System Corporation, Eden Prairie, MN, USA).

## 3. Results and Discussion

### 3.1. General Characterization of Chitin Derivatives: C_2_CHNC and C_12_CHNC

The preparation of PLA-based nanocomposite films with chitin nanocrystals as reinforcing nanofiller requires chemical modification of the nanofillers’ surface. Two sets of functionalized chitin nanocrystals (C_2_CHNC and C_12_CHNC, Figure 1) were prepared using heterogeneous conditions to increase the hydrophobic surface character, and thus improve the dispersability into the PLA matrix.

**Figure 2:**
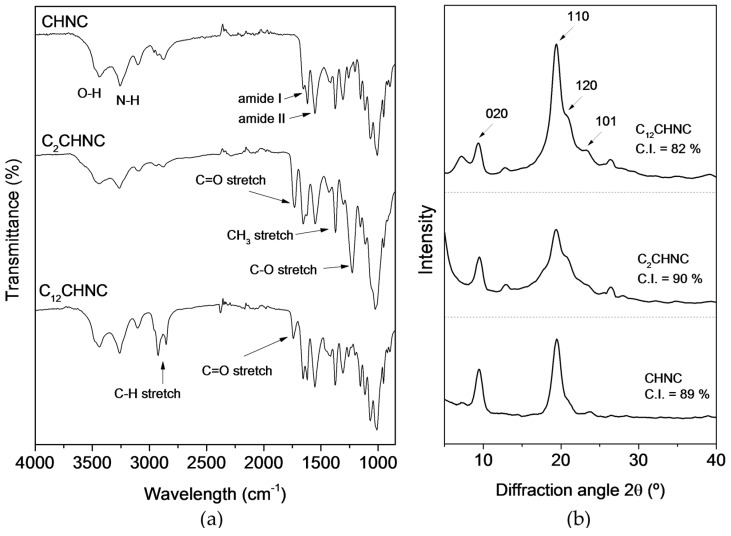
(**a**) ATR-FTIR spectra; and (**b**) X-ray diffraction of CHNC and acylated chitin nanocrystals (C_2_CHNC and C_12_CHNC).

Figure 2a displays ATR-FTIR spectrum of CHNC and its derivatives, i.e., C_2_CHNC (modified with anhydride acetic) and C_12_CHNC (modified with fatty acid). The success of the modifications was clearly confirmed, mainly based on the emergence of a new band at around 1735 cm^−1^, assigned to the carbonyl ester group stretching mode (C=O, Figure 2a) display in the C_2_CHNC and C_12_CHNC spectra [41,45,46,47]. This peak is smaller in the case of the nanocomposite films prepared with C_12_CHNC, possibly due to a smaller extent of the surface substitution. An increase in the intensity of the C–H band in the range 2860–2900 cm^−1^ arising from the aliphatic acid chain was also observed in the case of C_12_CHNC sample [48,49]. In all spectra the characteristic absorption bands of α-chitin i.e., at 1555 cm^−1^ ascribed to NH bending (amide II), at 1619 and 1655 cm^−1^ corresponding to carbonyl stretching bands (amide I) and at 3200 and 3400 cm^−1^ of N–H and O–H stretching band, respectively [50,51] are present. Nonetheless, a decrease in intensity of the broad band at about 3400 cm^−1^, in particular for C_2_CHNC sample, was observed indicating the substitution of hydroxyl groups by acetyl groups. This decrease and no disappearance of O–H stretching bands on the functionalized samples suggests that: (1) the modification has taken place on the surface of the chitin nanocrystals remaining intact inside hydroxyl group; and (2) an incomplete substitution of all of the surface OH groups [47,52].

The crystal structure of CHNC and its derivatives was analyzed by X-ray diffraction (Figure 2b). The four typical crystalline reflections of α-chitin at 9.5°, 19.5°, 21.0° and 23.5° indexed as 020, 110, 120 and 101 crystallographic planes, with a basal spacing of 9.31, 4.55, 4.25 and 3.80 Å, respectively, were observed [43,53,54]. The stronger reflections were clearly observed at 9.5° and 21.0° and the lower reflections at higher 2θ values. CHNC presented a crystallinity index of 89%. The crystalline structure of the functionalized chitin nanocrystals remained the same as CHNC and the C.I. were found to be 90% and 82% for C_2_CHNC and C_12_CHNC, respectively. The decrease of the crystallinity of C_12_CHNC is mainly due to the destruction of the crystalline regions of the nanocrystals, which led to a reduction of nanoparticle length (confirmed by AFM, see below). The fact that, modified chitin nanocrystals showed the same diffraction pattern than unmodified chitin, suggest that the modification took place at the surface of the nanocrystals and on the more accessible amorphous domains.

**Figure 3:**
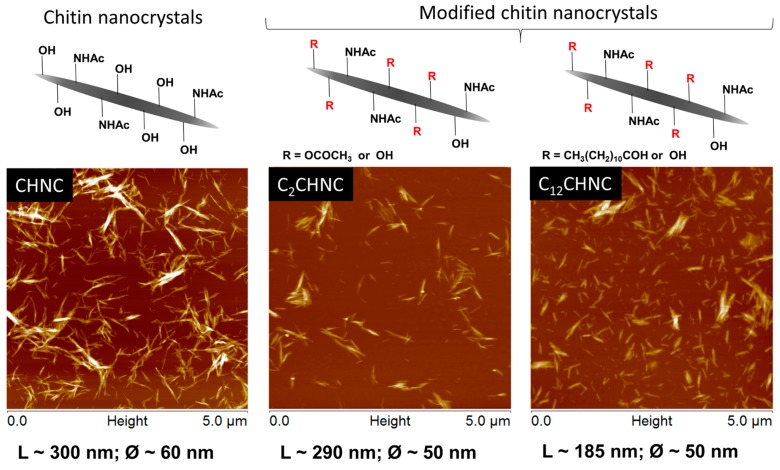
AFM height images of CHNC, C_2_CHNC and C_12_CHNC (L, length; and Ø, width).

This data corroborate with the sizes of CHNC and functionalized chitin nanocrystals that were roughly estimated using AFM imaging (Figure 3) and were found to be approximately 300, 290 and 185 nm in length (L) for CHNC, C_2_CHNC and C_12_CHNC, respectively. Actually, after the heterogeneous surface modification, the ensuing C_12_CHNC showed an important decrease of their length, justified by the partial detachment of the modified fibrils from the crystalline structure [55]. No significant changes were observed in the width (Ø) of the samples after the reactions. The typical rod-like morphology of the samples remained after the chemical modification.

The results have demonstrated that the modification was essentially limited to the nanocrystals’ surface or to the more accessible amorphous chitin domains, not affecting significantly the crystalline regions of the inner layers in the nanocrystals.

**Figure 4:**
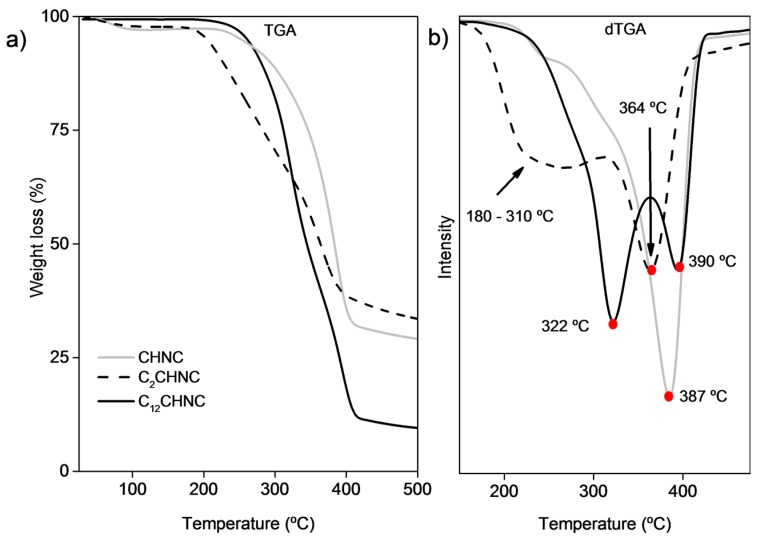
TGA (**a**); and dTGA (**b**) profiles of CHNC, C_2_CHNC and C_12_CHNC.

The thermal behavior of the CHNC before and after the reactions was evaluated by thermogravimetric analysis (Figure 4 and Table 2). Derivative TGA (dTGA) tracing of CHNC showed one weight-loss-step with a maximum degradation step at 387 °C associated with the degradation of chitin molecules. The thermal degradation profile of C_2_CHNC showed to be less stable, since they started to decompose at lower temperatures. Derivative TGA curves of C_2_CHNC displayed two degradation steps: the first is a wide degradation that started at around 180 until 310 °C; and the second step occurs at 364 °C. These thermal decomposition behaviors derived from chitin diacetate and chitin [45]. This slight decrease in thermal stability of CHNC is commonly found in surface-acetylated chitin. The derivative TGA curve of C_12_CHNC displayed two separate degradation steps at 322 °C and 390 °C associated to the degradation of esterified chitin fraction and unmodified chitin fraction, respectively. Interestingly, a decrease of water content in the C_12_CHNC samples was observed, suggesting that the modification of CHNC made them more hydrophobic [56,57].

**Figure 5:**
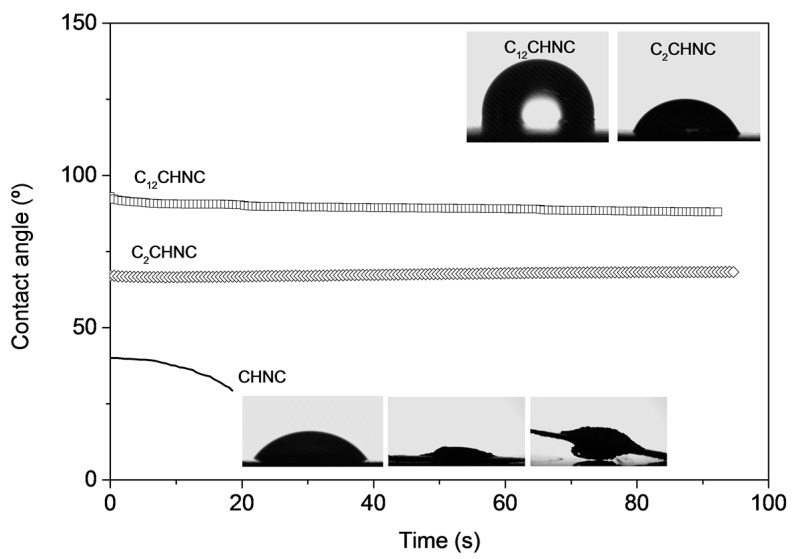
Dynamic water contact angle of CHNC and functionalized C_2_CHNC and C_12_CHNC and image of the water drop on the surface of the respective pellet.

Figure 5 displays the profile obtained for the dynamic water contact angle measurements placed on the surface of the CHNC pellets, before and after the surface chemical modifications with acetic anhydride and dodecanoyl chloride acid. Whereas the unmodified CHNC showed the typical high water affinity associated with a fast decrease in the initially low contact angle (55°) [58,59], modified C_2_CHNC and C_12_CHNC samples showed a higher (68.5° and 95.6°, respectively ) and constant contact angle [47]. The drop contact angle remained constant during the time (Figure 5) as a result of the hydroxyl groups’ replacement by hydrophobic chains (acetyl groups and dodecanoyl chains) on the surface of acylated chitin nanocrystals. As expected, C_12_CHNC displayed the larger value of contact angle (95.6°) due to its long aliphatic chain. The surface energy components (polar γ_sp_, dispersive γ_sd_ and total γ_s_) of C_12_CHNC samples were determined and are listed in Table 3. The decrease in the polar component (γ_sp_) values from 16.3 (CHNC) to 0.7 mJ m^−2^ (C_12_CHNC) confirmed the chemical modification on the CHNC surface.

### 3.2. Characterization of PLA-Based Nanocomposites (PLA/C_2_CHNC and PLA/C_12_CHNC)

#### 3.2.1. Optical Properties of the Nanocomposites

**Figure 6:**
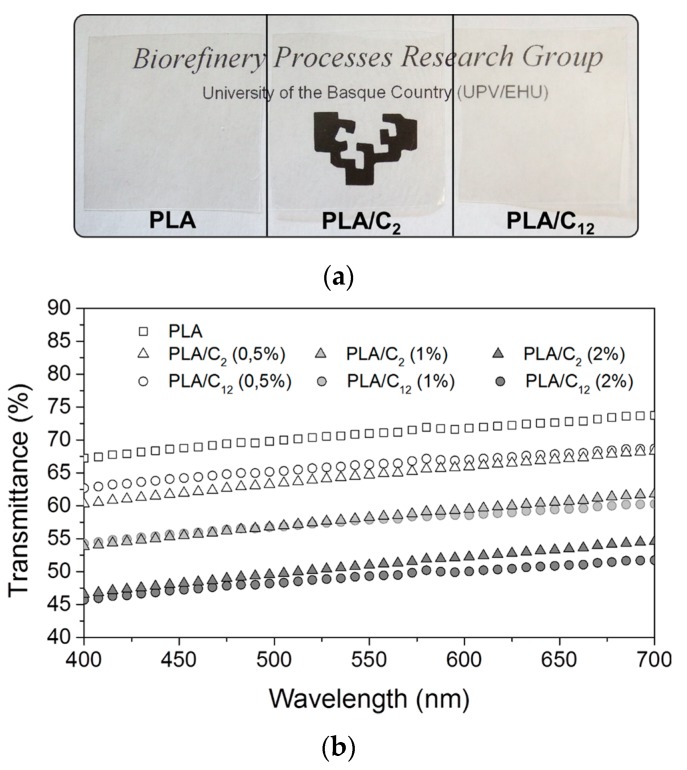
Photographs of PLA, PLA/C_2_0.5 and PLA/C_12_0.5 nanocomposite films (**a**); and transmittance traces of PLA film and PLA/C_2_ and PLA/C_12_ nanocomposite films (**b**).

One of the important parameters in packaging is the transparency, thus the light transmittance of the films was measured from 700 to 400 nm (Figure 6). As listed in Figure 6b, the addition of nanofillers in the matrix affected the transmittance of the final nanocomposites, decreasing its values gradually with the increment of chitin nanocrystals in the matrix. However, the materials are still translucent, as shown in Figure 6a. The transmittance (measured for specimens with a thickness of approximately 0.07 mm) at 600 nm was about 72% for PLA, 66% for PLA/C_2_0.5 and 67% for the PLA/C_12_0.5 nanocomposites (Figure 6b). These results indicate that the transmittance is not affected by the kind of modified CHNC.

#### 3.2.2. Thermal and Mechanical Properties

**Figure 7:**
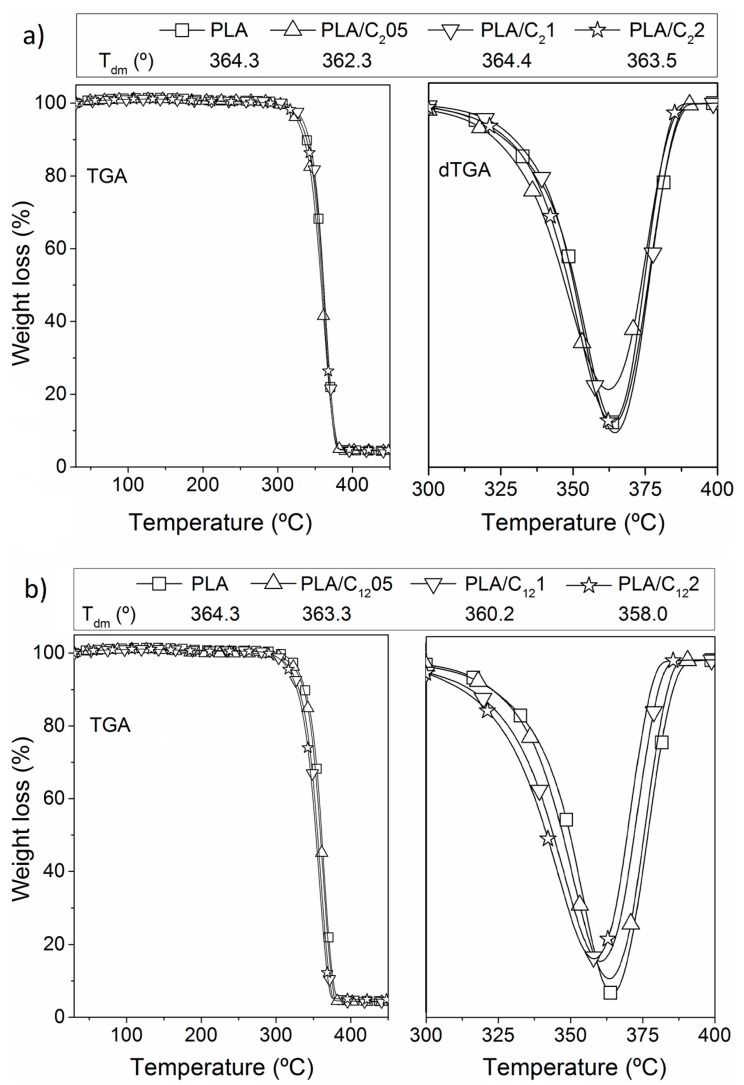
Themogravimetric analysis of: PLA/C_2_ nanocomposite (**a**); and PLA/C_12_ nanocomposite (**b**).

Like for the modified CHNC, the thermal stability and degradation profiles of the PLA nanocomposite materials were assessed by thermogravimetry (Table 1). The TGA profile of the PLA film exhibits a typical single weight-loss step, with a maximum decomposition rate at 364 °C. The thermal decomposition process of all the PLA/C_2_CHNC (Figure 7a) and PLA/C_12_CHNC (Figure 7b) nanocomposites also presented a single weight loss step profile. The thermal stability profiles of all samples were very similar without important changes, contrarily with Guan et al. work [15] where the thermal degradation of PLA bionanocomposites with 2% of chitin nanocrystals decreased about 25 °C.

**Figure 8:**
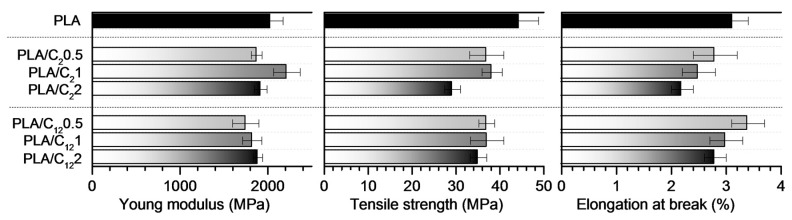
Mechanical properties of PLA films and PLA nanocomposites in terms of Young’s modulus, tensile strength and elongation at break.

The mechanical properties of PLA films and nanocomposite films were evaluated in terms of Young’s modulus, tensile strength and elongation at break (Figure 8). The unfilled PLA films showed characteristic values in Young’s modulus at around 2 GPa, in tensile strength of 44 MPa and elongation at break of 3.1% [60,61]. Contrary to previous works [20,62,63] related to the incorporation of CHNC into PLA matrices, in the present work, a minor loss in modulus and tensile strength was observed when the modified CHNC were incorporated in the PLA matrices. This effect was associated to the C_2_CHNC and C_12_CHNC aggregation. However, PLA-based nanocomposites prepared with other type of nanofillers (i.e., nanocrystalline cellulose, and nanoclays) also showed no significant effect on the mechanical properties of the final materials [14]. Regarding elongation at break, characteristic decrease of this parameter was observed in all materials due to the incorporation of crystal nanofillers.

#### 3.2.3. Contact Angles and Surface Energy of the PLA-Based Nanocomposites

The contact angle values of PLA nanocomposites are summarized in Table 3. Regarding the contact angles of the PLA/C_2_ nanocomposites, the data were similar to those of PLA film. However, the enhancement in the hydrophobic character of the C_12_CHNC after esterification was clearly demonstrated by the superior water contact angle from 89 to 96° for the PLA/C_12_ nanocomposites compared with 81° for the PLA films. The surface energy of the PLA/C_12_ nanocomposites were calculated using other liquids besides water with different polarities—diiodomethane and ethylenglycol (Table 3).

The surface energy of PLA/C_12_ (32 mJ/m^2^) was lightly superior to PLA film (27 mJ/m^2^) because of the high values of the dispersive component surface energy of the modified CHNC. No significant differences on the surface energy were found regarding the amount of modified chitin in the PLA nanocomposites.

#### 3.2.4. Barrier Properties: Water Vapor Transmission Tests

**Figure 9:**
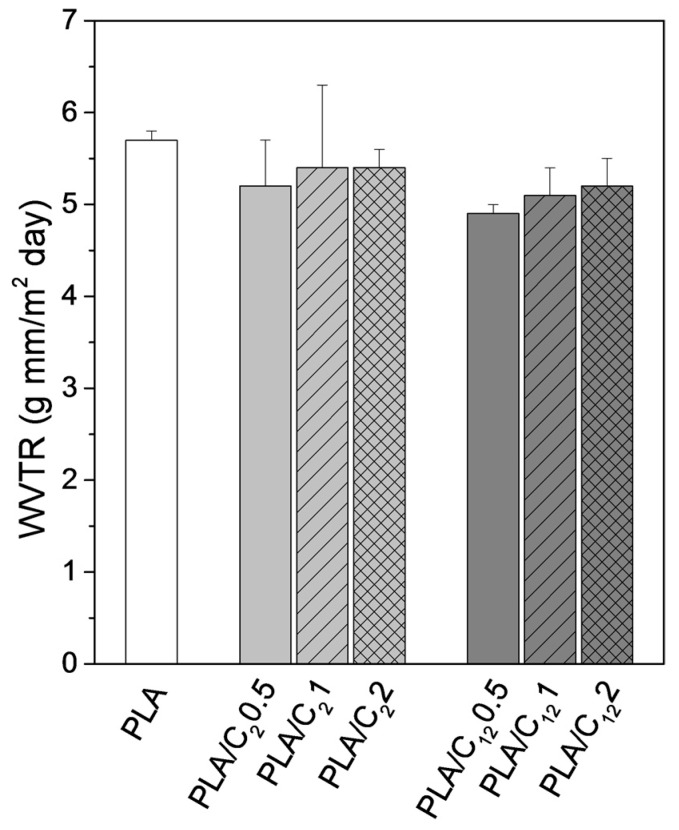
WVTR of PLA films and PLA nanocomposites.

The water vapor transmission rate (WVTR) of PLA-based nanocomposites was analyzed because of the potential use of these materials in food packaging. The assessment of this parameter is important because of the role of water in microbial development and deteriorative effects. In general, the data listed in Figure 9 demonstrate a slight decrease in the WVTR, which is directly associated with the incorporation of hydrophobic groups on the surface of the CHNC. The range of values is in accordance with those found in previous works [43]. As expected, the decrease in the WVTR is more evident for the PLA-based nanocomposites prepared with the CHNC modified with fatty aliphatic chains. The amount (0.5%, 1% and 2%) of both sets of CHNC did not significantly affect the WVTR. These results are consistent with the water contact angles measurements.

#### 3.2.5. Study of the Fungal Inhibition

The main purpose of fungal inhibition study towards *A. niger* was to analyze the potential use of PLA-chitin based nanocomposites films for active food packaging applications, since it is a determinant factor for this industry. The study was performed using *A. niger* since it is known as a widespread contaminant of food namely fruits and vegetables. Moreover, some of *A. niger* strains has been linked to a wide array of human health problems [64].

**Figure 10:**
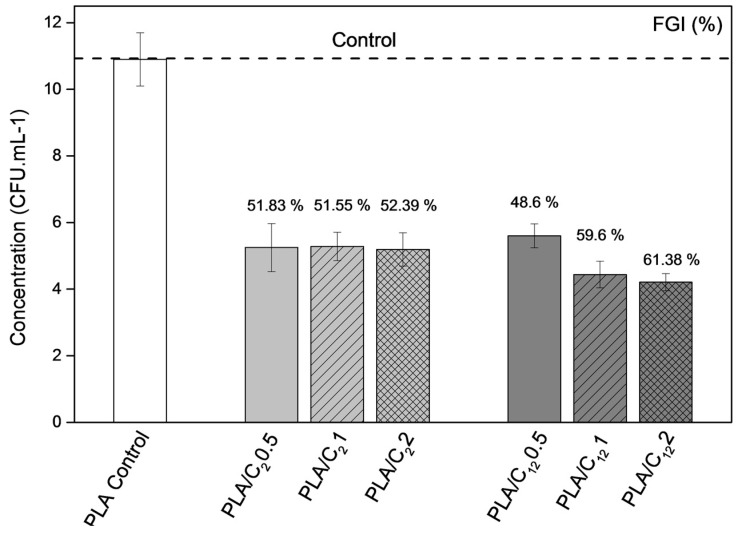
Antifungal activity against *A. niger* of PLA film (control sample), PLA/C_2_ and PLA/C_12_ bionanocomposite films. The value on the top of each bar corresponds to the fungal growth inhibition (FGI, %). Error bar corresponds to standard deviation (SD, *n* = 4).

The antifungal and antimicrobial effect of chitin and chitin nanofillers has been previously reported [34,65,66,67]. Figure 10 shows the number of colony forming units per milliliter and the fungal growth inhibition (FGI, %) for the antifungal activity of the PLA film (reference) and the PLA-based nanocomposite films after seven days of *A. niger* incubation at 25 °C. The data showed that the incorporation of modified CHNC considerably decreased the fungal activity of all materials. Nevertheless, the two sets of nanocomposite films presented distinguish behaviors: (1) PLA/C_2_ films showed a constant fungal growth inhibition of approximately 50% independently of the C_2_CHNC concentration; and (2) the PLA/C_12_ materials presented an increase of the fungal growth inhibition from 48% to 61% with the incorporation of C_12_CHNC with respect to the reference material (PLA film).

These results are in agreement with the results reported earlier concerning the incorporation of chitin nanocrystals and/or nanofibers in other polymeric matrices [36,37,65,66,67].

## 4. Conclusions

Chitin nanocrystals were successfully modified via acylation using anhydride acetic and dodecanoyl chloride acid. Poly(lactic acid)-based nanocomposite films were prepared by extrusion/compression approach using different concentrations of both sets of modified CHNC followed by their evaluation for packaging applications. The data demonstrated that both sets of modified CHNC act as functional agents, i.e., they slightly enhanced the hydrophobic character of the materials and improved the antifungal activity. The different CHNC amounts incorporated in the PLA matrix have no detrimental effect on the mechanical properties in tension and light transmittance, compared to neat PLA. It was also found that the nanocomposite films prepared with the CHNC modified with dodecanoyl chloride acid presented better properties to act as active food packaging.

## Figures and Tables

**Table 1:** Identification of the PLA/C_2_CHNC and PLA/C_12_CHNC samples.

Sample Identification	Acylation	CHNC (wt %)
PLA	–	–
PLA/C_2_0.5	Acetic anhydride	0.5
PLA/C_2_1	Acetic anhydride	1
PLA/C_2_2	Acetic anhydride	2
PLA/C_12_0.5	Dodecanoyl chloride acid	0.5
PLA/C_12_1	Dodecanoyl chloride acid	1
PLA/C_12_2	Dodecanoyl chloride acid	2

**Table 2:** Degradation temperatures of CHNC, C_2_CHNC, C_12_CHNC and PLA-based nanocomposites.

Sample	Temp. Main Degradation (°C)	Temp. Second Degradation (°C)
CHNC	387.1	
C_2_CHNC	364.3	180–310
C_12_CHNC	390.2	322.8
PLA	364.3	
PLA/C_2_0.5	362.3	
PLA/C_2_1	364.4	
PLA/C_2_2	363.5	
PLA/C_12_0.5	363.3	
PLA/C_12_1	360.2	
PLA/C_12_2	358.0	

**Table 3:** Contact angle and surface energy components (polar γ_sp_, dispersive γ_sd_ and total γ_s_) values of the samples.

Samples	Contact Angles (°)			(γ_sp_)	(γ_sd_)	(γ_s_)
	Water	Diiodomethane	Ethylene Glycol	(mJ/m^2^)		
CHNC	55.0 ± 2.0	45.5 ± 1.0	26.6 ± 0.8	16.3 ± 1.1	33.4 ± 0.4	49.7 ± 1.6
C_12_CHNC	95.6 ± 1.9	53.3 ± 1.5	81.4 ± 2.1	0.7 ± 0.2	28.0 ± 0.5	28.7 ± 0.7
PLA	81.3 ± 1.9	82.5 ± 1.3	58.8 ± 1.8	10.5 ± 1.0	16.5 ± 0.3	27.0 ± 1.4
PLA/C_12_0.5	88.8 ± 0.9	53.7 ± 2.1	65.2 ± 2.5	2.2 ± 0.2	30.2 ± 0.2	32.4 ± 0.5
PLA/C_12_1	89.1 ± 2.1	53.6 ± 1.2	66.4 ± 1.9	2.1 ± 0.5	30.1 ± 0.5	32.2 ± 1.1
PLA/C_12_2	95.6 ± 1.1	53.5 ± 0.8	69.0 ± 1.1	0.8 ± 0.1	31.1 ± 0.3	31.8 ± 0.4
